# Association between exposure to traffic-related air pollution and pediatric allergic diseases based on modeled air pollution concentrations and traffic measures in Seoul, Korea: a comparative analysis

**DOI:** 10.1186/s12940-020-0563-6

**Published:** 2020-01-14

**Authors:** Kyung-Duk Min, Seon-Ju Yi, Hwan-Cheol Kim, Jong-Han Leem, Ho-Jang Kwon, Soyoung Hong, Kyoo Sang Kim, Sun-Young Kim

**Affiliations:** 1 Department of Public Health Science, Graduate School of Public Health, Seoul National University, Seoul, Republic of Korea; 2 Institute of Health and Environment, Seoul National University, Seoul, Republic of Korea; 3 Institute of Endemic Diseases, Medical Research Center, Seoul National University College of Medicine, Seoul, Republic of Korea; 4 Department of Occupational and Environmental Medicine, Inha University School of Medicine, Incheon, Republic of Korea; 5 Department of Preventive Medicine, Dankook University College of Medicine, Chungnam, Republic of Korea; 6 Department of Environmental Health Research, Seoul Medical Center, Seoul, Republic of Korea; 7 Department of Cancer Control and Population Health, Graduate School of Cancer Science and Policy, National Cancer Center, Goyang-si, Gyeonggi-do Republic of Korea

**Keywords:** Air pollution, Atopic eczema, Individual-level exposure, Pediatric allergic diseases, Traffic

## Abstract

**Background:**

Pediatric allergic diseases are a major public health concern, and previous studies have suggested that exposure to **t**raffic-related air pollution (TRAP) exposure is a risk factor. These studies have typically assessed TRAP exposure using traffic measures, such as distance to major roads, or by modeling air pollutant concentrations; however inconsistent associations with pediatric allergic diseases have often been found. Using road proximity and density, we previously found an association between TRAP and atopic eczema among approximately 15,000 children living in Seoul, Korea, heavily populated and highly polluted city in which traffic is a major emission source. We aimed to conduct a parallel analysis using modeled air pollution concentrations and thus examine the consistency of the association. Specifically, we examined the associations of individual-level annual-average concentrations of NO_2_, PM_10_, and PM_2.5_ with symptoms and diagnoses of three pediatric allergic diseases including asthma, allergic rhinitis, and atopic eczema.

**Methods:**

The study population included 14,614 children from the Seoul Atopy Friendly School Project Survey in Seoul, Korea, in 2010. To assess individual exposures to TRAP among these children, we predicted annual-average concentrations of NO_2_, PM_10_, and PM_2.5_ at the children’s home addresses in 2010 using universal kriging and land use regression models along with regulatory air quality monitoring data and geographic characteristics. Then, we estimated odds ratios (ORs) of the three allergic diseases for interquartile increases in air pollution concentrations after adjusting for individual risk factors in mixed effects logistic regression.

**Results:**

Symptoms and diagnoses of atopic eczema symptoms showed an association with NO_2_ (OR = 1.07, 95% confidence interval = 1.02–1.13; 1.08, 1.03–1.14) and PM_10_ (1.06, 1.01–1.12; 1.07, 1.01–1.13). ORs of PM_2.5_ were positive but not statistically significant (1.01, 0.95–1.07; 1.04, 0.98–1.10). No association was found between asthma and allergic rhinitis, although PM_2.5_ showed a marginal association with allergic rhinitis.

**Conclusions:**

Our consistent findings regarding the association between TRAP and the prevalence of atopic eczema using traffic measures and surrogate air pollutants suggested the effect of TRAP on children’s health. Follow-up studies should elucidate the causal link, to support subsequent policy considerations and minimize adverse health effects in children.

## Background

Pediatric allergic diseases, including asthma, allergic rhinitis, and atopic eczema, are major public health concerns, owing to their adverse physical and psychological effects, which result in a social and economic burden [1]. Although previous reports from developed countries showed that their prevalence had reached a plateau, a global trend for the increase in the prevalence is now commonly reported [2–5].

Previous studies have shown that geographical [6], genetic [7], and other environmental factors [8], can confer risk factors of the pediatric allergic diseases; however, air pollution has also been suspected of contributing to the current upward trend in allergic diseases [9, 10]. Several epidemiological studies have reported associations of exposure to particulate matter (PM) ≤ 10 and ≤ 2.5 μm in diameter (PM_10_ and PM_2.5_) and nitrogen dioxide (NO_2_) with asthma [11], rhinitis [12] and eczema [13]. Several possible biological mechanisms have also been suggested. Previous toxicological studies have reported that PM can cause epithelial barrier dysfunction and increase oxidative stress, which may increase allergic responses [14]. NO_2_ can modify immune responses to various allergens [15, 16] possibly by acting as an adjuvant, leading to sensitization or potentiation of inflammatory effects on an allergen challenge as discussed in a previous study [17].

In particular, understanding the association of allergic diseases with a specific source of air pollution is important because this enables the relevant authorities to establish a clear target of intervention for reducing air pollution and preventing subsequent adverse health effects. Specifically, exposure to traffic-related air pollution (TRAP) has become of particular interest because traffic-related interventions, such as implementation of diesel retrofit programs, restriction of high emission vehicles, and accommodation of energy-efficient vehicles, are highly practical options for reducing air pollution in many countries [18, 19].

Exposure to TRAP has been assessed either by direct measures of traffic or measurement of air pollutant concentrations [20]. Each approach has strengths and limitations. For direct traffic measures, although a few studies have used traffic volume estimated for road segments, these data on traffic volume are largely unavailable. Most studies have instead adopted road density or proximity computed based on road network data [21, 22]. Road density is quantified using the sum of major road lengths within a circular buffer area from a target location, whereas road proximity is computed as the distance to the nearest major road. These traffic measures are considered to estimate the air pollution directly caused by traffic. However, these estimates may not represent the extent of exposure among people; it is therefore difficult to interpret results regarding toxicity of TRAP. In addition, such quantification may not be accurate as the proximity to or density of major roads does not necessarily indicate the amount of traffic on these roads. However, the concentrations of traffic-related air pollutants may be used to characterize exposure to TRAP, including traffic emissions, and could thus be used to assess toxicity among people. However, traffic-related air pollutants may also include emissions from sources other than traffic, which makes it difficult to isolate the impact of traffic. Some studies have used both air pollutants and traffic measures, with inconsistent findings regarding their association with allergic diseases [23, 24].

A study using both direct traffic measures and air pollution concentrations can enhance our understanding in the association between TRAP and allergic diseases. Our recent epidemiological study assessed TRAP using road proximity and density measurements in a heavily populated and highly polluted city, Seoul, Korea. We found an association between TRAP and atopic eczema in our study, which included approximately 15,000 children [25]. Using the same study population, exposure to TRAP could be assessed by modeled air pollution concentrations, instead of traffic measures. The comparison of two sets of health analysis results from two different measures for TRAP can provide strong evidence of the association between TRAP and allergic diseases. In particular, traffic conditions and related air pollution characteristics in Seoul provide a unique opportunity to investigate the association of TRAP. People in Seoul tend to live near arterial roads with heavy congestion for easy access to transportation; the average of road density within 300 m from children’s residences in our previous study was 7200m^2^ [25]. NO_2_ and PM, suggested as traffic-related air pollutants in previous studies, also showed their relationships with traffic in Seoul. There was high correlation between traffic volume estimates and NO_2_ concentrations in Seoul [26, 27]. A previous review of source apportionment studies indicated that a major source of PM_10_ and PM_2.5_ in Seoul is traffic [28].

We aimed to investigate the association between exposure to TRAP and the prevalence of three pediatric allergic diseases using individual-level concentrations of three traffic-related air pollutants among about 15,000 children living in Seoul, Korea and to compare the results with those obtained from the analyses based on traffic measures in our previous study. The three air pollutants examined were NO_2_, PM_10_, and PM_2.5_, and we assessed the prevalence of three pediatric allergic diseases, namely, asthma, allergic rhinitis, and atopic eczema.

## Methods

### Study population

We used survey data of the Seoul Atopy Friendly School Project. The survey was conducted from May to October in 2010, and included 31,576 children from 136 elementary schools and 34 kindergartens. Participating schools and kindergartens were located across all 25 districts in Seoul; every district has at least two schools or kindergartens. Details of the survey have been previously described [29].

**Fig. 1:**
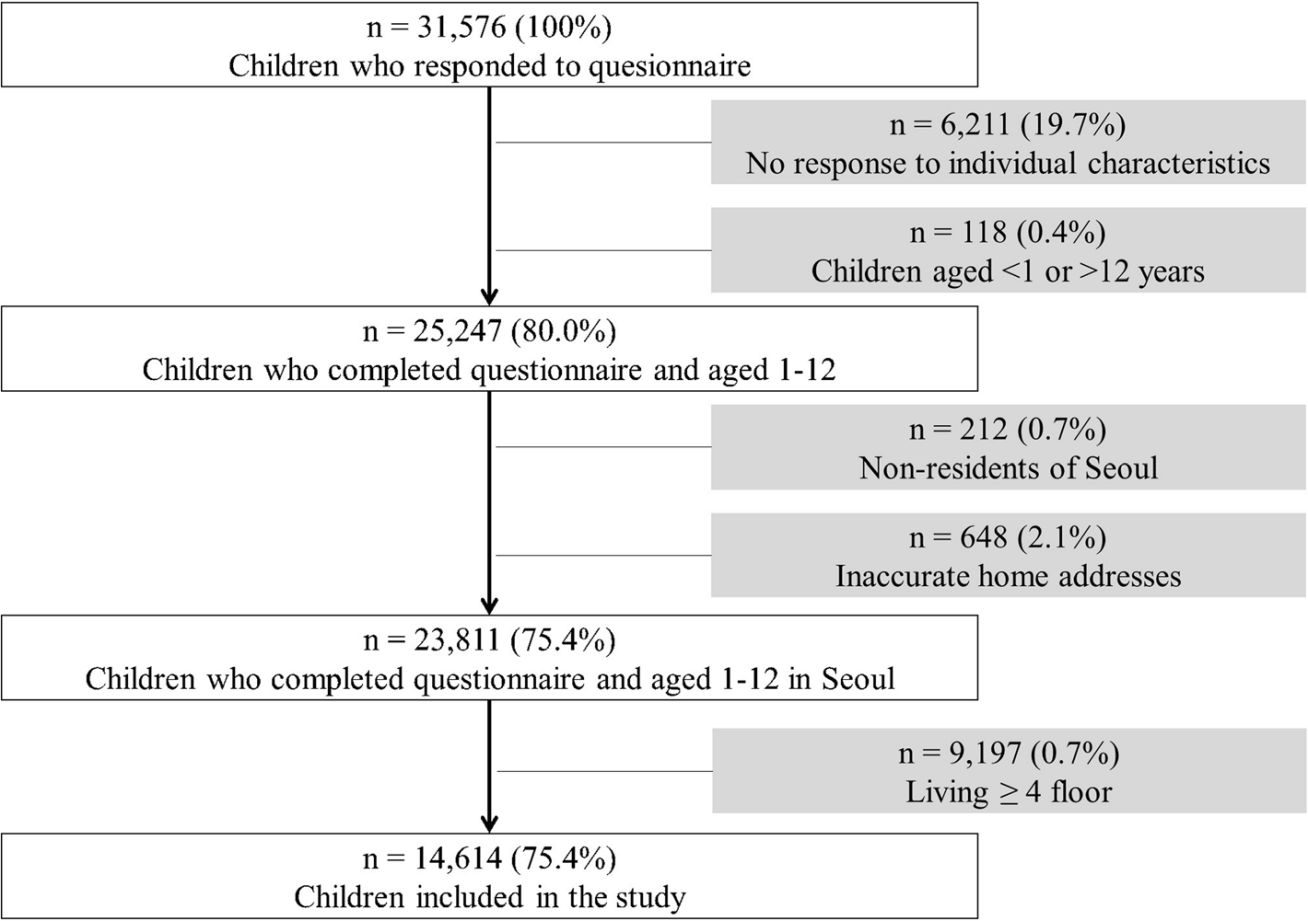
Schematic diagram of selecting the 14,614 study population from the Seoul Atopy Friendly School Project Survey participants in Seoul, Korea, for 2010

We excluded 16,386 children who did not meet the inclusion criteria [25] (Fig. 1). We excluded participants who had incomplete data, were aged < 1 or > 12 years, lived outside of Seoul, or had inaccurate home addresses. We also excluded participants living at the fourth floor level or higher because air pollution exposure in these individuals may be less accurately represented by regulatory monitoring data collected at sites that are mostly located at ground level or on the top of public buildings. Finally, we included 14,614 children in the analysis.

### Assessment of air pollution exposure

To assess children’s individual exposures to NO_2_, PM_10_, and PM_2.5_, we predicted annual average concentrations, from January to December in 2010, at the children’s home addresses using universal kriging for NO_2_ and PM_10_, and land use regression for PM_2.5_, based on regulatory monitoring data and geographic variables (Additional file 1: Figure S1).

For air pollution regulatory monitoring data, we obtained hourly measurements of NO_2_, PM_10_, and PM_2.5_ at 37 regulatory monitoring sites in Seoul during 2010 from the National Institute of Environmental Research (NIER) [30]. The 37 monitoring sites consisted of 25 urban background and 12 urban roadside sites. The urban background sites were mostly located in residential areas with no pollution sources nearby. Each of the 25 districts (24.21 km^2^ area, on average) in Seoul had at least one urban background site in 2010. The urban roadside sites are located next to large, busy roads for monitoring TRAP. Using hourly air pollution concentrations, we estimated daily average concentrations, excluding days with < 18 hourly measurements (25%). We then computed annual means at sites that met the inclusion criteria. The exclusion criteria used to select sites with representative annual averages were sites with > 91 missing days (25%), > 45 consecutive missing days, or < 10 months without at least one daily average per month [31]. All 37 monitoring sites met the criteria.

**Fig. 2:**
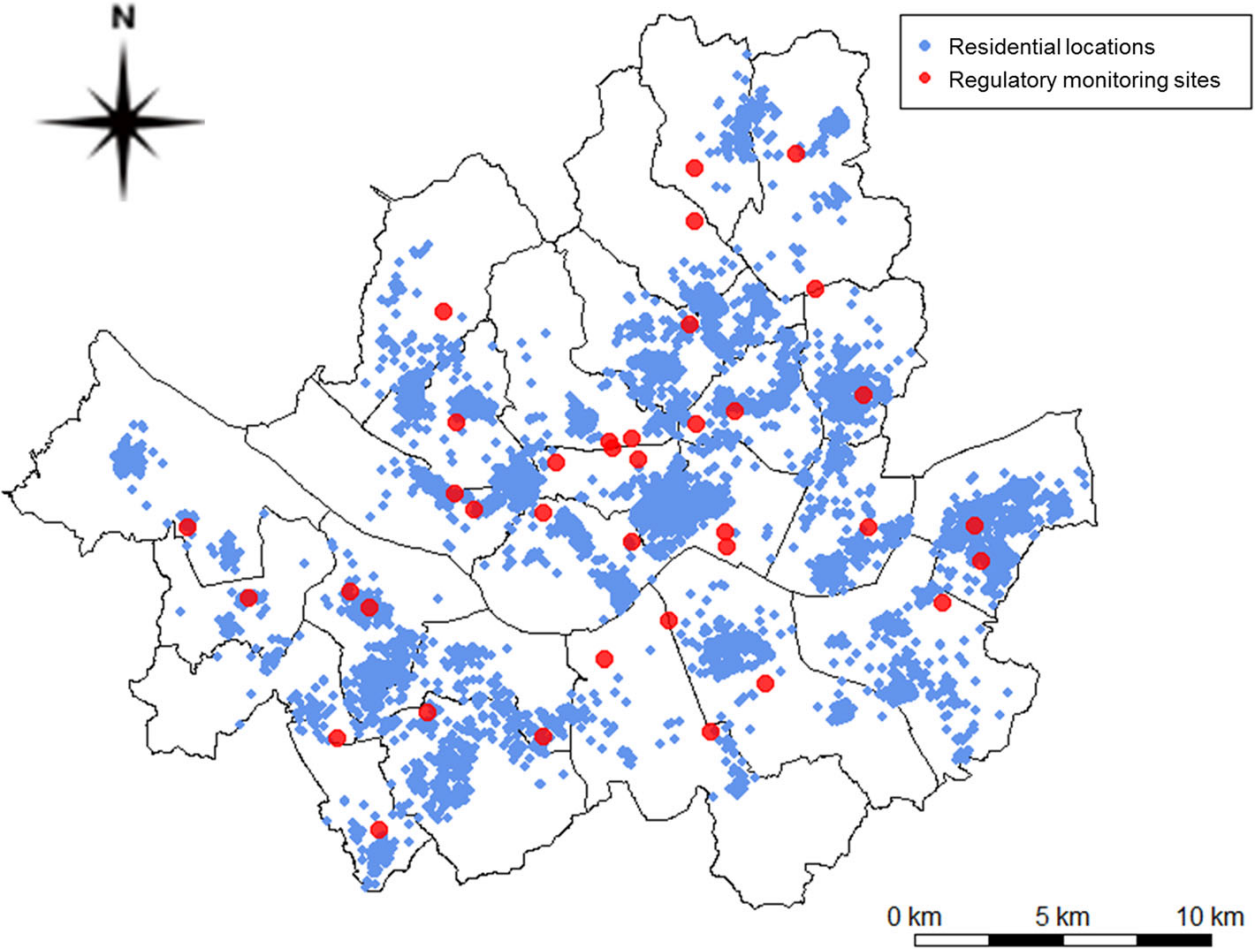
Map of home addresses of 14,614 children of the Seoul Atopy Friendly School Project Survey and 37 air pollution regulatory monitoring sites in Seoul, Korea, for 2010

We computed 313 geographic variables at the 37 regulatory monitoring sites as well as the homes and schools of the 14,614 children who participated in the Seoul Atopy Friendly School Survey. We obtained coordinates of regulatory monitoring sites from the NIER and the geocoded addresses of children’s homes and schools using Geocoder-Xr (Geoservice, Seoul, Korea) (Fig. 2). The geographic variables included potential air pollution sources for eight categories of traffic, demographic characteristics, land use, transportation facilities, physical geography, emissions, vegetation, and altitude [32]**.**

Using regulatory monitoring data and geographic variables, we predicted annual average concentrations of NO_2_ and PM_10_ at children’s home and school addresses using universal kriging prediction models. In this approach, it is assumed that air pollution concentrations are composed of geographic predictors and spatial correlation [33]. We estimated a few summary predictors with more than 300 geographic variables using partial least squares (PLS) [34]. For PM_2.5_, because the PLS summary predictors showed an overfitting pattern, we instead applied land use regression, including five geographic variables selected using forward selection [35]. These models showed good performance for all three pollutants (cross-validated R^2^s of 0.83, 0.82, and 0.69 for NO_2_, PM_10_, and PM_2.5_, respectively), indicating good prediction ability.

### Allergic diseases

We identified children with current and doctor-diagnosed allergic disorders for asthma, allergic rhinitis, and atopic eczema based on the questionnaire of the Seoul Atopy Friendly School Project Survey, translated from the International Study of Asthma and Allergies in Children (ISAAC) questionnaire [36]. The ISAAC questionnaire is considered a standardized tool for identifying allergic symptoms and diagnoses [37–39], and has been widely applied in many epidemiological studies [6–8]. The questions used in the present study for defining current symptoms of asthma, rhinitis, and eczema were as follows [29]: “Has your child had wheezing or whistling in the chest in the past 12 months?”; “In the past 12 months, have you had a problem with sneezing or a runny or blocked nose when you did not have a cold or the flu?”; and “Has your child had an itchy rash at any time in the past 12 months?”. We also used the following specific questions to define three doctor-diagnosed allergic outcomes: “Has your child ever been diagnosed with asthma by a medical doctor?”; “Has your child ever been diagnosed with allergic rhinitis by a medical doctor?”; and “Has your child ever been diagnosed with atopic eczema by a medical doctor?”. Children with symptoms of allergic diseases were identified as those who answered yes to the questions regarding symptoms within the previous 12 months, whereas children diagnosed with allergic diseases were identified as those for whom the answer was yes to questions addressing both on symptoms and diagnoses. The parents or guardians completed the written questionnaire. The survey also included socio-demographic information and other individual characteristics related to the incidence of allergic disorders, such as breast feeding and adiposity.

This study was approved by the institutional review board (IRB) at the Seoul National University (IRB approval number: SNU IRB No. E1503/002–004).

### Statistical analysis

We conducted logistic regression analysis to examine the associations between predicted individual-level annual-average air pollution concentrations and allergic diseases in children. The associations were assessed for each of the 18 pairs, comprising the three air pollutants (NO_2_, PM_10_, and PM_2.5_) and six allergic outcomes, including symptoms and doctor-diagnosed asthma, allergic rhinitis, and atopic eczema. Effect estimates were presented as odds ratios (ORs) and 95% confidence intervals (95% CIs) for an interquartile range (IQR) increment in each pollutant concentration. We developed three confounder models for each analysis, to progressively adjust for socio-economic status and/or disease-related characteristics. In model 1, we included demographic factors only (age and sex). We added categorized variables for household income, body mass index (BMI), and breast feeding in model 2. Household income was categorized into three levels based on tertiles: high, middle, and low. Children’s BMI was also classified into three categories based on percentiles given the sex and age: underweight (< 25%), normal (25–85%), and obese (≥ 85%). Breast feeding duration was categorized as follows: ≥ 12, 4–11, and ≤ 3 months. As our primary model, in model 3 we added random effects at the school and residential district area, to adjust for unmeasured area-level confounding and to account for correlation of children’s allergic disorders within the same school and residential district area. For the residential district area, we categorized all 25 districts in Seoul into eight areas by aggregating 2–4 nearby districts. We determined residential district areas via combining of the 25 districts in this way because some districts consist of much smaller numbers of schools and children than others. For asthma and allergic rhinitis, we restricted the analyses to children aged ≥6 years, based on previously reported difficulties in identifying the clinical diagnosis of these two allergic diseases and in distinguishing these from other infectious diseases in preschool-aged children [40–42].

In addition, we compared our exposure estimates and health analysis results with those based on our previous study using road network variables [25]. These road variables represented road density and proximity computed as the sum of lengths of major roads multiplied by road widths and the number of lanes, and the distance to the closest major road within 300 m from children’s homes, respectively. Major roads were defined as national highways or roads with six lanes or more [32]. This comparison was done based on the same input data and health analysis models including the identical covariates.

Our sensitivity analysis included analysis using an alternative individual-level exposure metric that incorporated air pollution exposure at the children’s schools or kindergartens. We predicted annual average concentrations at school addresses using the same prediction methods as those used for homes. We recomputed individual air pollution concentrations by averaging the predicted concentrations at homes and schools using a ratio of 2:1 because the average operating hours of kindergartens are reported as 7 h and 34 min [43] and elementary school students remain at school for 8 h, on average [44]. As another sensitivity analysis, we examined the association of asthma by presence of allergic and non-allergic diseases, as the prevalence of asthma comprises a large proportion of non-allergic cases and the association of TRAP was modified by presence of allergic diseases [17]. We conducted our analysis for asthma in children with a diagnosis of allergic rhinitis or atopic eczema and compared the results with those in children who did not have these diagnoses.

We also performed stratified analyses according to regional and household socioeconomic status (SES) and examined whether the association varies by the two types of SES. Based on the rate of financial independence in the 25 districts during 2010, obtained from the Seoul Open Data [45], we computed the rates for eight residential district areas as regional SES, and categorized as follows: high (≥ 70%), middle (40–70%), and low (< 40%) groups. Household SES was divided into three categories based on tertiles of household income in the survey. We investigated the associations in each of the nine groups jointly categorized according to the three regional and three household SES groups.

All statistical analyses conducted in this study were performed using R version 3.5.1 (The R Foundation for Statistical Computing, Vienna, Austria; https://www.r-project.org/).

## Results

Table 1 shows general characteristics of the 14,614 children selected as the study population from the Seoul Atopy Friendly School Project Survey. A 49.8% of the children was male and 25.1% were aged ≤6 years. Obese children were made up 5.2% of the group and half of the children had been breastfed for < 4 months.

**Table 1:** Individual characteristics of 14,614 children in the Seoul Atopy Friendly School Project Survey in Seoul, Korea, for 2010

Variable	Level	Total N	%	Prevalence^a^					
				Eczema	%	Asthma	%	Rhinitis	%
		14,614	100	2323	15.9	1171	8.0	5286	36.2
Sex	Male	7337	49.8	1140	15.5	711	9.7	2966	40.4
	Female	7277	50.2	1183	16.3	460	6.3	2320	31.9
Age (year)	1–3	1293	8.8	218	16.9	231	17.9	362	28.0
	4–6	2389	16.3	439	18.4	267	11.2	879	36.8
	7–9	5404	37.0	888	16.4	378	7.0	2061	38.1
	10–12	5528	37.8	778	14.1	295	5.3	1984	35.9
BMI	Underweight	2029	13.9	284	14.0	166	8.2	835	41.2
	Normal	11,826	80.9	1920	16.2	918	7.8	4170	35.3
	Obese	759	5.2	119	15.7	87	11.5	281	37.0
Breast feeding (month)	≤3	7975	54.6	1110	13.9	590	7.4	2950	37.0
	4–11	3836	26.2	630	16.4	317	8.3	1329	34.6
	≥12	2803	19.2	583	20.8	264	9.4	1007	35.9
Household income	Low	2638	18.1	471	17.9	226	8.6	832	31.5
	Middle	6467	44.3	1086	16.8	526	8.1	2343	36.2
	High	5509	37.7	766	13.9	419	7.6	2111	38.3
Regional income	Low	5036	34.5	760	15.1	306	6.1	1928	38.3
	Middle	6903	47.2	1101	15.9	618	9.0	2470	35.8
	High	2675	18.3	462	17.3	247	9.2	888	33.2

^a^ Prevalence was identified based on symptoms

The period prevalence within a year for the three allergic diseases based on symptoms was 15.9, 8.0, and 36.2% for eczema, asthma, and rhinitis, respectively. Male children had higher prevalence of asthma (9.7%) and rhinitis (40.4%) than females (6.3 and 31.9%, respectively). Children aged < 7 years showed higher prevalence of eczema (17.8%) and asthma (13.5%) but lower prevalence of rhinitis (33.7%) than older children (15.7, 6.3, and 38.0%, respectively). Obese children had higher prevalence of asthma (11.5%) than underweight children (8.2%), but rhinitis was more prevalent in underweight children (41.2%) than obese ones (37.0%). Children who had been breastfed for a short period showed lower prevalence of eczema (13.9%) than those breastfed for over a year (20.8%). Children from low-income households had higher prevalence of eczema (17.9%) and asthma (8.6%) than those from high-income households (13.9 and 7.6%, respectively), whereas children living in high SES regions had higher prevalence of these allergic diseases (17.3 and 9.2%) than in low SES regions (15.1 and 6.1%). For children with prevalent allergic diseases based on a doctor’s diagnosis, the patterns of individual characteristics were generally consistent with those with children who had symptoms (Additional file 2; Table S1). One exception was that much fewer children aged ≤6 years had asthma and allergic rhinitis. This indicates the possibility of overestimated prevalence among young children based on questions addressing symptoms, justifying our restriction of the analyses to children aged ≥6 years. The means of predicted annual average concentrations of NO_2_, PM_10_, and PM_2.5_ at children’s homes were 35.99 ppm, 49.67 μg/m^3^, and 25.30 μg/m^3^, respectively (Table 2).

**Table 2:** Summary statistics of annual average concentrations of PM_10_, PM_2.5_, and NO_2_ predicted at home addresses of 14,614 children in the Seoul Atopy Friendly School Project Survey in Seoul, Korea, for 2010

Exposure	Mean	SD	Min	Median	Max
Home only					
PM_10_ (μg/m^3^)	49.67	3.2	39.07	49.4	66.3
PM_2.5_ (μg/m^3^)	25.3	3.0	18.42	25.03	50.89
NO_2_ (ppm)	35.99	5.8	22.52	34.71	75.24
Home and school (2:1)^a^					
PM_10_ (μg/m^3^)	49.36	2.3	42.29	49.17	61.1
PM_2.5_ (μg/m^3^)	25.13	2.2	19.38	24.94	41.69
NO_2_ (ppm)	35.6	4.1	26.93	34.91	63.76

^a^ Weighted means of predicted air pollution concentrations at homes and schools using the ratio of 2:1

**Fig. 3:**
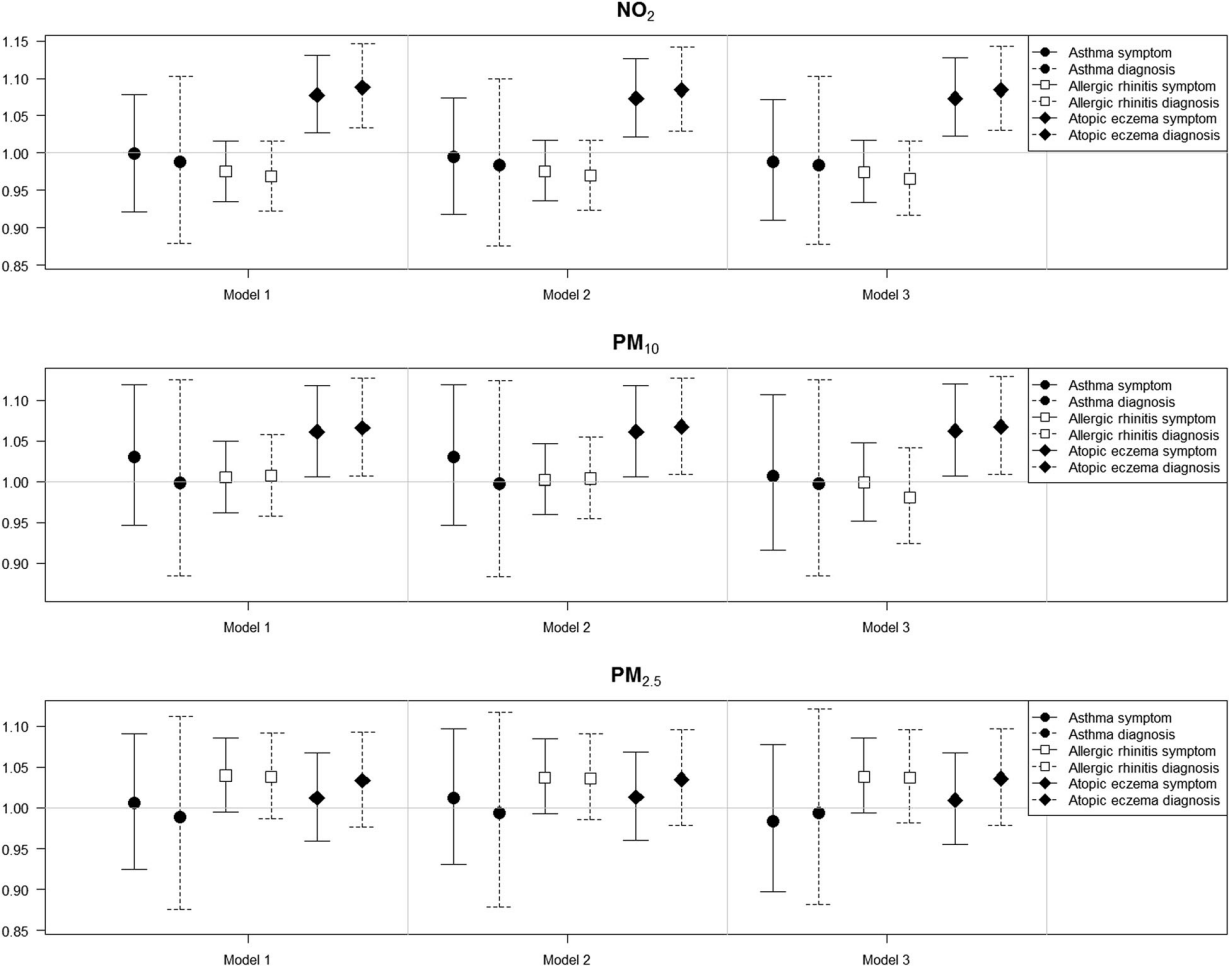
Odds ratios and 95% confidence intervals of symptoms and doctor-diagnoses of three allergic diseases for interquartile increases in individual-level annual average concentrations of NO_2_, PM_10_, and PM_2.5_ (6.46 ppm, 3.80 μg/m^3^, and 3.63 μg/m^3^, respectively) in 14,614 children of the Seoul Atopy Friendly School Project Survey in Seoul, Korea, for 2010

For TRAP and allergic diseases, we found associations of eczema symptoms with NO_2_ (OR = 1.07, 95% CI = 1.02–1.13) and with PM_10_ (1.06, 1.01–1.12) (Fig. 3, Additional file 3: Table S2). These associations were consistent with a diagnosis of atopic eczema, with slightly higher ORs for diagnosis than symptoms (NO_2_: 1.08, 1.03–1.14; PM_10_: 1.07, 1.01–1.13). All three confounder models showed consistent associations. PM_2.5_ showed positive but non-significant ORs for both symptom and diagnosis (1.01, 0.95–1.07; 1.04, 0.98–1.10, respectively). There were no associations of asthma and allergic rhinitis for all three pollutants and two types of allergic diseases. However, allergic rhinitis based on both symptom and diagnosis was marginally associated with PM_2.5_ (1.04, 0.99–1.09; 1.04, 0.98–1.10, respectively).

In a comparison with the results obtained in our previous study [25], using road density and proximity for TRAP, the two sets of analyses using different exposure metrics showed noticeably consistent results. Yi et al. (2017) found negative associations of atopic eczema with the distance to major roads and positive associations with the sum of major road lengths [25]. Both studies consistently found that increased TRAP was associated with increased prevalence of atopic eczema but not asthma and allergic rhinitis (Additional file 4: Table S3). Annual-average concentrations of the three air pollutants at children’s homes were negatively correlated with the distance to the nearest major roads and positively correlated with the sum of major road lengths within 300 m from the children’s homes (Additional files 5 and 6: Figures S2 and S3).

When we recomputed children’s exposures to TRAP by including air pollution concentrations at their schools in addition to their homes, we found associations of NO_2_ with atopic eczema symptom and diagnosis; ORs for these (model 3 OR = 1.06, 95% CI = 1.01–1.12; 1.07, 1.01–1.13) were slightly lower than ORs based on children’s home addresses only in our main analysis (Additional file 7: Figure S4, Additional file 8: Table S4). ORs for PM_10_ were positive but showed marginal significance (1.05, 0.99–1.11; 1.05, 0.99–1.12, respectively). In the analysis for asthma using children with and without a diagnosis of allergic rhinitis or atopic eczema, effect estimates were mostly close to the null and non-significant, without any clear pattern of differences between the two groups. (Additional files 9 and 10: Table S5 and S6).

In the stratified analysis by regional and household SES jointly, the highest ORs for atopic eczema were found for low regional and middle household SES and for high regional and low household SES, across all pollutants. Whereas ORs were generally low in the group with high regional and high household SES for all three allergic diseases, low regional and low household SES did not always yield higher ORs than in the other groups. In addition, we found statistically significant associations of PM_2.5_ with both symptom and diagnosis of allergic rhinitis (1.23, 1.07–1.40; 1.20, 1.03–1.39) in children from the group of middle household and low regional SES. These high effect estimates possibly resulted in the marginal association of PM_2.5_ with allergic rhinitis for all children (Additional files 11, 12, and 13: Figures S5, S6, and S7).

## Discussion

As a follow-up study of previous findings regarding the association between TRAP and the prevalence of atopic eczema using traffic estimates in about 15,000 children residing in Seoul, Korea, we investigated the association with three allergic outcomes by identifying TRAP exposure as individual-level concentrations of NO_2_, PM_10_, and PM_2.5_. We found consistent findings for the associations of symptom-based as well as doctor-diagnosed atopic eczema with NO_2_ and PM_10_. Our findings suggest that the consistent associations observed using both metrics, characterized by road network data and air pollutants, confirm TRAP as a risk factor of allergic responses, at least for atopic eczema, in a large city where traffic is a major pollution source.

Our results confirmed the association between TRAP and atopic eczema, using two different forms of exposure to TRAP and two types of outcomes including symptom and diagnosis. Concentrations of NO_2_, PM_10_, and PM_2.5_ predicted at children’s homes were highly correlated with the sum of major road lengths and distance to the closest major road used in our previous study. The associations with atopic eczema between the two types of exposure metrics were also consistent. This consistency indicates that the main contributors of NO_2_, PM_10_, and PM_2.5_ in Seoul are traffic emissions, as supported in a previous review of source apportionment studies in South Korea [26]. We also showed consistent findings between the two types of measurements for the allergic diseases; symptom and diagnosis. Symptom measures are likely to detect prevalence with high sensitivity and low specificity, resulting in over-estimation of the true prevalence. In contrast, diagnosis measures may have low sensitivity and high specificity, leading to under-estimation. There were much fewer children aged ≤6 years with asthma and rhinitis symptoms than with asthma and rhinitis diagnoses in our study, which also indicates the possibility of overestimated prevalence in young children based on symptom questions. To avoid the impact of this misclassification, we restricted our analysis for asthma and allergic rhinitis to children aged ≥6 years. In addition, we used the diagnosis measure as a complementary outcome. Our consistent results indicate that the influence of potential misclassification would be minimal.

Although a clear biological explanation for the association between air pollution and atopic eczema has not been established, the possible pathogenesis has been suggested [46]. Direct contact of air pollutants with the skin could produce oxidative stress and damage to structures in the epidermis, resulting in skin barrier dysfunction. Moreover, inhalation of air pollutants via the pulmonary system could overactivate the expression of IL-4, contributing to the development of atopic eczema. One study investigated the effect of diesel exhaust particle (DEP); they also suggested that DEP was associated with atopic dermatitis in mouse models [47].

We did not find an association between PM_2.5_ and allergic outcomes, although PM_2.5_ yielded positive effect estimates and was highly correlated with the sum of and distance to major roads. Previous epidemiological findings have also suggested that NO_2_ and PM_10_ could affect development and exacerbation of atopic eczema [13, 48] However, no association was found between PM_2.5_ and atopic eczema in a prospective birth cohort study [49]. The differing findings between PM_10_ and PM_2.5_ may be due to the differences in chemical composition and emission sources. Future studies should be conducted to confirm these different patterns across pollutants [50].

We did not find significant associations for asthma and allergic rhinitis. A recent review study showed associations between outdoor NO_2_ and pediatric asthma development and symptoms of wheezing [51]; experimental evidences has also supported the relationship between PM and asthma [52]. Although further investigations are needed to elucidate these discrepancies, various etiologies of asthma other than exposure to air pollution may explain these differences [53, 54]. Particularly genetic predisposition and/or secondary smoking, which were not included in this study, could act as confounders and may lead to different results without adjustment. In addition, non-allergic components of asthma possibly included in the asthma prevalence in our study might have affected the results of no association. Previous studies showed higher effect estimates of TRAP in people diagnosed with allergic diseases than in those who were never diagnosed [17] and significant estimates only in individuals with allergic asthma [55]. However, our sensitivity analysis restricted to children diagnosed with allergic rhinitis or eczema also showed no associations. Our findings of no association between TRAP and allergic rhinitis, although there was a marginal association for PM_2.5_, were also inconsistent with previous epidemiological and toxicological findings [56, 57]. Rhinitis has a dynamic natural course, indicating that the symptoms of rhinitis are not persistent and could disappear within 2 years [58]. Considering the cross-sectional nature of this study, the association with allergic rhinitis could have been difficult to capture.

In our sensitivity analysis, incorporating school exposures tended to alter the association towards the null. These attenuated effect estimates may be due to reduced exposure variability, because the same air pollution concentrations predicted at a school were assigned to all children at the same school. In addition, most geocoded addresses of schools were the centroids of relatively large areas that included school buildings, playgrounds, and other facilities. This may have resulted in exposure misclassification and affected subsequent health effect estimation.

We found that the probability of having allergic diseases tended to be high in children with low household or low regional SES when combined with middle household or high regional SES, respectively, although effect estimates were not statistically different. Effect estimates in these groups were even higher than those found in the group of low regional and low household SES. This pattern indicates the possibility of an interactive impact of deprivation between household and regional levels. The higher risk estimates found in children from lower-income families was consistent with previous findings [59], which can be explained by co-existing risk factors in lower-income groups, such as low quality diet and living in a stressful environment [59].

There were several limitations in this study. First, measurement errors of outcome might have affected our findings. The data obtained from the self-reported questionnaires could overestimate prevalence. Recent validation studies of the ISAAC questionnaires in South Korea reported overestimated prevalence for allergic rhinitis [60] and atopic eczema [61]. In particular, this overestimation could have been greater as the questions regarding symptoms are not allergy-specific. However, consistent findings with respect to the association with TRAP in our study between symptoms and doctor-diagnoses based on allergic-specific questions indicate that the impact of misclassification could be negligible. Second, we did not include other potential confounders, such as family characteristics and indoor environments, which were unavailable in our study. Future studies should include family characteristics related to smoking status, allergic history, and educational attainment of parents, and the presence of other identified allergens based on companion animals, chemicals, or house dust in children’s homes, preschools, and/or schools. As the Seoul Atopy Friendly School Project Survey did not include a skin prick test, information on the presence of sensitization to common allergens was also unavailable in this study. Further studies including information of sensitization and important allergens should be conducted to confirm our findings. The absence of genetic predisposition should be also considered in future research. Third, as motor vehicles largely produce fine exhaust particles such as ultra-fine particles and PM_2.5_, our inclusion of PM_10_ in TRAP might be uncommon. However, relatively large particles attributed to rising dust from vehicles travelling on roads may also contribute to children’s allergic symptoms. The high correlation of PM_10_ with road density and proximity in our study as well as the high contribution of traffic to PM_10_ according to a previous review study of source apportionment in Seoul [26] may support this application in a highly populated metropolitan area, such as Seoul. Lastly, the causal relationship between TRAP exposure and allergic disease development was unclear in the present study, especially in children aged over 5 years. Because our exposure assessment was limited to 1-year averages in 2010 and did not incorporate previous exposures such as information about relocation, our findings suggest a relationship with persistence of allergic diseases rather than their development. Future studies based on birth cohorts should investigate the impact on incidence or development of allergic diseases.

## Conclusion

In this study, we found evidence of the association between traffic-related air pollution and prevalence of atopic eczema in children, based on a large and representative population in a highly urbanized city in which traffic is a major pollution source. In particular, consistent findings observed using both traffic measures and air pollution concentrations confirmed the association. As people in megacities tend to live near large, busy roads for easy access to transportation, the number of the affected children and the level of risk would be enormous. Future studies based on extended data including early air pollution exposure and exposure to common allergens should further investigate the associations with the incidence and development of allergic diseases, and provide policy recommendations to minimize the adverse health effects of traffic-related air pollution particularly for children living in large cities.

## Supplementary information

**Additional file 1: Figure S1.** Data preprocessing and prediction procedure for assessing individual-level concentrations of PM_10_, PM_2.5_, and NO_2_ at 14,614 children’s home and school addresses in the Seoul Atopy Friendly School Project Survey in Seoul, Korea, for 2010.

**Additional file 2: Table S1.** Individual characteristics of 14,614 children in the Seoul Atopy Friendly School Project Survey in Seoul, Korea, for 2010.

**Additional file 3: Table S2.** Odds ratios and 95% confidence intervals of symptoms and doctor-diagnoses of three allergic diseases for interquartile increases in individual-level annual average concentrations of NO_2_, PM_10_, and PM_2.5_ (6.46 ppm, 3.80 μg/m^3^, and 3.63 μg/m^3^, respectively) in 14,614 children of the Seoul Atopy Friendly School Project Survey in Seoul, Korea, for 2010.

**Additional file 4: Table S3.** Comparison of odds ratios and 95% confidence intervals of three allergic diseases for individual-level air pollution concentrations with those for traffic measures (Yi et al. 2017) based on the identical population of 14, 614 children of the Seoul Atopy Friendly School Survey in Seoul, Korea, for 2010.

**Additional file 5: Figure S2.** Scatter plots between predicted annual-average concentrations of NO_2_, PM_10_, or PM_2.5_ in 2010 and distances to the closest major roads (Yi et al. 2017) at home addresses of 14,614 children in the Seoul Atopy Friendly School Project Survey in Seoul, Korea, for 2010 (red lines for non-linear relationships estimated by locally-weighted smoothing).

**Additional file 6: Figure S3.** Scatter plots between predicted annual-average concentrations of NO_2_, PM_10_, or PM_2.5_ in 2010 and total length of major roads within 300 m buffers (Yi et al. 2017) at home addresses of 14,614 children in the Seoul Atopy Friendly School Project Survey in Seoul, Korea, for 2010 (red lines for non-linear relationships estimated by locally-weighted smoothing).

**Additional file 7: Figure S4.** Odds ratios and 95% confidence intervals of symptoms and doctor-diagnoses of three allergic diseases for interquartile increases in individual-level annual average concentrations of NO_2_, PM_10_, and PM_2.5_ (4.7 ppm, 2.85 μg/m^3^, and 2.69 μg/m^3^, respectively) in 14,614 children at homes and schools from the Seoul Atopy Friendly School Project Survey in Seoul, Korea, for 2010.

**Additional file 8: Table S4.** Odds ratios and 95% confidence intervals of symptoms and doctor-diagnoses of three allergic diseases for interquartile increases in individual-level annual average concentrations of NO_2_, PM_10_, and PM_2.5_ (4.7 ppm, 2.85 μg/m3, and 2.69 μg/m3, respectively) in 14,614 children at homes and schools from the Seoul Atopy Friendly School Project Survey in Seoul, Korea, for 2010.

**Additional file 9: Table S5.** Odds ratios (ORs) and 95% confidence intervals (95% CIs) of asthma symptoms and diagnoses for individual-level concentrations of NO_2_, PM_10_, PM_2.5_ by children with and without allergic rhinitis and atopic eczema diagnoses.

**Additional file 10: Table S6.** Odds ratios (ORs) and 95% confidence intervals (95% CIs) of asthma symptoms and diagnoses for individual-level concentrations of NO_2_, PM_10_, PM_2.5_ by children with and without presence of at least one of allergic rhinitis and atopic eczema diagnosis.

**Additional file 11: Figure S5.** Odds ratios and 95% confidence intervals of symptoms and doctor-diagnoses of three allergic diseases for interquartile increases in individual-level annual average concentrations of NO_2_ (6.46 ppm) in 14,614 children at homes stratified by regional and household socioeconomic status from the Seoul Atopy Friendly School Project Survey in Seoul, Korea, for 2010.

**Additional file 12: Figure S6.** Odds ratios and 95% confidence intervals of symptoms and doctor-diagnoses of three allergic diseases for interquartile increases in individual-level annual average concentrations of PM_10_ (3.80 μg/m^3^) in 14,614 children at homes stratified by regional and household socioeconomic status from the Seoul Atopy Friendly School Project Survey in Seoul, Korea, for 2010.

**Additional file 13: Figure S7.** Odds ratios and 95% confidence intervals of symptoms and doctor-diagnoses of three allergic diseases for interquartile increases in individual-level annual average concentrations of PM_2.5_ (3.63 μg/m^3^) in 14,614 children at homes stratified by regional and household socioeconomic status from the Seoul Atopy Friendly School Project Survey in Seoul, Korea, for 2010.

## Data Availability

The datasets analyzed in this study are available from the corresponding author sykim@ncc.re.kr on reasonable request.

## Abbreviations

DEP: Diesel Exhaust Particle
ISAAC: International Study of Asthma and Allergies in Children
NIER: National Institute of Environmental Research
NO_2_: Nitrogen Dioxide
PLS: Partial Least Squares
PM: Particulate Matter
SES: Socioeconomic Status
TRAP: Traffic Related Air Pollution
