# Supplementary material for: Association between exposure to traffic-related air pollution and pediatric allergic diseases based on modeled air pollution concentrations and traffic measures in Seoul, Korea: a comparative analysis
Source: Environ Health. 2020 Jan 14;19:6. doi: 10.1186/s12940-020-0563-6 (PMC6961284; doi:10.1186/s12940-020-0563-6)
Supplement: Supplementary file 1 — Additional file 1: Figure S1. Data preprocessing and prediction procedure for assessing individual-level concentrations of PM10, PM2.5, and NO2 at 14,614 children’s home and school addresses in the Seoul Atopy Friendly School Project Survey in Seoul, Korea, for 2010. [file 12940_2020_563_MOESM1_ESM.docx]

**
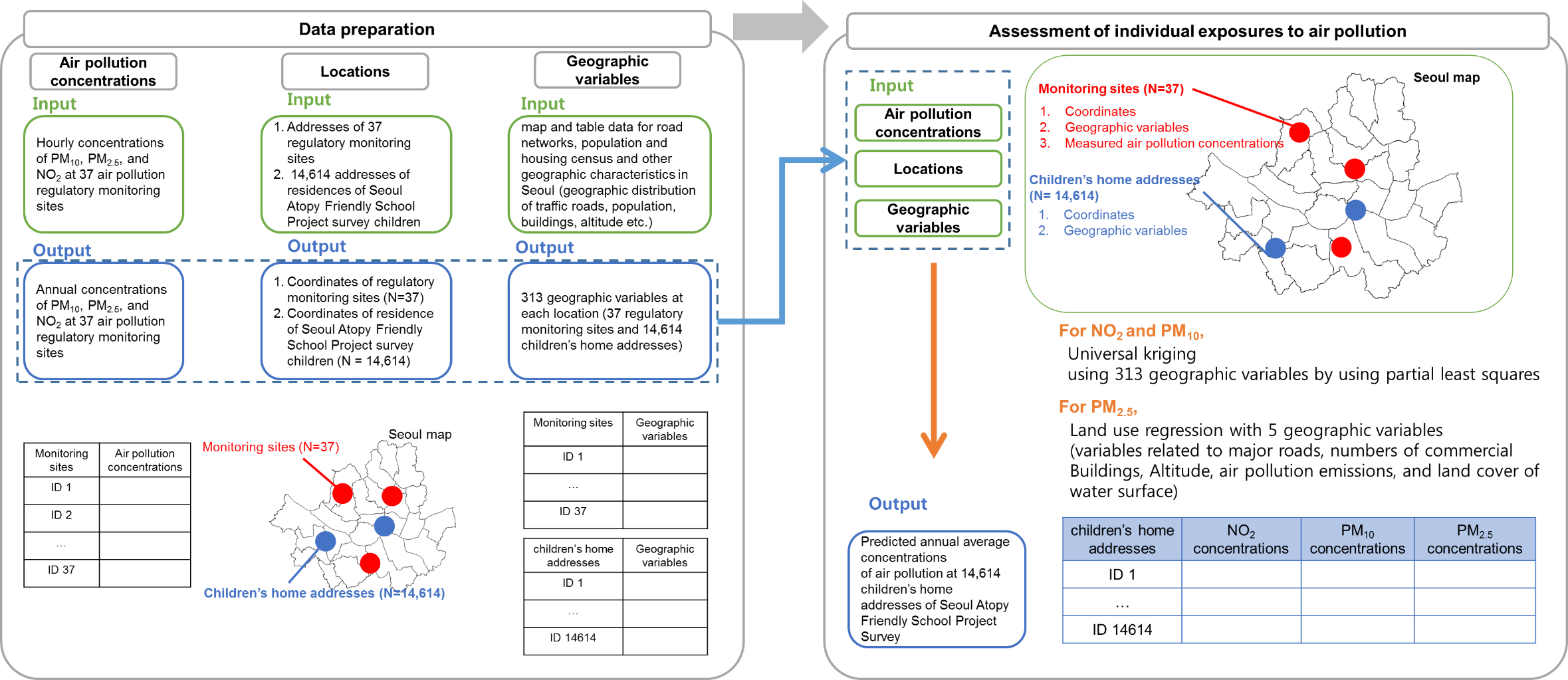
**

**Figure S1 Data preprocessing and prediction procedure for assessing individual-level concentrations of PM10, PM2.5, and NO2 at 14,614 children’s home and school addresses in the Seoul Atopy Friendly School Project Survey in Seoul, Korea, for 2010**
