# Supplementary material for: Association between exposure to traffic-related air pollution and pediatric allergic diseases based on modeled air pollution concentrations and traffic measures in Seoul, Korea: a comparative analysis
Source: Environ Health. 2020 Jan 14;19:6. doi: 10.1186/s12940-020-0563-6 (PMC6961284; doi:10.1186/s12940-020-0563-6)
Supplement: Supplementary file 2 — Additional file 2: Table S1. Individual characteristics of 14,614 children in the Seoul Atopy Friendly School Project Survey in Seoul, Korea, for 2010. [file 12940_2020_563_MOESM2_ESM.docx]

**Table S1 Individual characteristics of 14,614 children in the Seoul Atopy Friendly School Project Survey in Seoul, Korea, for 2010**

| **Variable** | **Level** | **Total N** | **%** | **Prevalence (diagnosis)^1^** | | | | | |
| --- | --- | --- | --- | --- | --- | --- | --- | --- | --- |
|  |  |  |  | **Eczema** | **%** | **Asthma** | **%** | **Rhinitis** | **%** |
|  |  | 14614 | 100 | 1972 | 13.5 | 460 | 3.2 | 3198 | 21.9 |
| **Sex** | |  |  |  |  |  |  |  |  |
|  | **Male** | 7337 | 49.8 | 970 | 13.2 | 292 | 4.0 | 1896 | 25.8 |
|  | **Female** | 7277 | 50.2 | 1002 | 13.8 | 168 | 2.3 | 1302 | 17.9 |
| **Age (year)** | |  |  |  |  |  |  |  |  |
|  | **1-3** | 1293 | 8.8 | 186 | 14.4 | 43 | 3.3 | 126 | 9.7 |
|  | **4-6** | 2389 | 16.3 | 368 | 15.4 | 85 | 3.6 | 421 | 17.6 |
|  | **7-9** | 5404 | 37.0 | 754 | 14.0 | 179 | 3.3 | 1350 | 25.0 |
|  | **10-12** | 5528 | 37.8 | 664 | 12.0 | 153 | 2.8 | 1301 | 23.5 |
| **BMI** | |  |  |  |  |  |  |  |  |
|  | **Underweight** | 2029 | 13.9 | 241 | 11.9 | 69 | 3.4 | 547 | 27.0 |
|  | **Normal** | 11826 | 80.9 | 1625 | 13.7 | 356 | 3.0 | 2491 | 21.1 |
|  | **Obese** | 759 | 5.2 | 106 | 14.0 | 35 | 4.6 | 160 | 21.1 |
| **Breast feeding (month)** | | |  |  |  |  |  |  |  |
|  | **≤3** | 7975 | 54.6 | 943 | 11.8 | 245 | 3.1 | 1825 | 22.9 |
|  | **4-11** | 3836 | 26.2 | 539 | 14.1 | 121 | 3.2 | 795 | 20.7 |
|  | **≥12** | 2803 | 19.2 | 490 | 17.5 | 94 | 3.4 | 578 | 20.6 |
| **Household income** | |  |  |  |  |  |  |  |  |
|  | **Low** | 2638 | 18.1 | 398 | 15.1 | 80 | 3.0 | 410 | 15.5 |
|  | **Middle** | 6467 | 44.3 | 911 | 14.1 | 212 | 3.3 | 1449 | 22.4 |
|  | **High** | 5509 | 37.7 | 663 | 12.0 | 168 | 3.1 | 1339 | 24.3 |
| **Regional income** | |  |  |  |  |  |  |  |  |
|  | **Low** | 5036 | 34.5 | 648 | 12.9 | 148 | 2.9 | 1234 | 24.5 |
|  | **Middle** | 6903 | 47.2 | 940 | 13.6 | 236 | 3.4 | 1469 | 21.3 |
|  | **High** | 2675 | 18.3 | 384 | 14.4 | 76 | 2.8 | 495 | 18.5 |

^1^ Prevalence was identified based on doctor-diagnosis
