# Supplementary material for: Association between exposure to traffic-related air pollution and pediatric allergic diseases based on modeled air pollution concentrations and traffic measures in Seoul, Korea: a comparative analysis
Source: Environ Health. 2020 Jan 14;19:6. doi: 10.1186/s12940-020-0563-6 (PMC6961284; doi:10.1186/s12940-020-0563-6)
Supplement: Supplementary file 4 — Additional file 4: Table S3. Comparison of odds ratios and 95% confidence intervals of three allergic diseases for individual-level air pollution concentrations with those for traffic measures (Yi et al. 2017) based on the identical population of 14, 614 children of the Seoul Atopy Friendly School Survey in Seoul, Korea, for 2010. [file 12940_2020_563_MOESM4_ESM.docx]

**Table S3 Comparison of odds ratios and 95% confidence intervals of three allergic diseases for individual-level air pollution concentrations with those for traffic measures (Yi et al. 2017) based on the identical population of 14, 614 children of the Seoul Atopy Friendly School Survey in Seoul, Korea, for 2010**

| **Outcome** | **Min. et al. 2019 (this study)** | | | **Yi et al (2017)*** | |
| --- | --- | --- | --- | --- | --- |
|  | **NO_2_** | **PM_10_** | **PM_2.5_** | **Distance to the major roads** | **Density of major roads** |
| **Atopic eczema** | **1.07 (1.02 – 1.13)** | **1.06 (1.01– 1.12)** | **1.01 (0.95 – 1.07)** | **≤150m 1.15 (1.01 – 1.32)**  **150-300m 1.17 (1.02 – 1.34)**  **300-500m 1.16 (1.01 – 1.33)**  **>500m 1.00** | **1.08 (1.01 – 1.15)** |
| **Asthma** | **0.99 (0.92 – 1.06)** | **1.00 (0.92 – 1.08)** | **0.98 (0.91 – 1.06)** | **≤150m 0.93 (0.78 – 1.11)**  **150-300m 1.11 (0.93 – 1.32)**  **300-500m 1.00 (0.83 – 1.20)**  **>500m 1.00** | **0.94 (0.86 – 1.03)** |
| **Allergic rhinitis** | **0.97 (0.94 – 1.01)** | **1.00 (0.95 – 1.04)** | **1.03 (0.94 – 1.01)** | **≤150m 0.97 (0.88 – 1.07)**  **150-300m 1.05 (0.95 – 1.16)**  **300-500m 1.00 (0.90 – 1.12)**  **>500m 1.00** | **0.97 (0.92 – 1.03)** |

* While this study assessed TRAP by using air pollution concentrations at children’s home addresses, Yi, et al. (2017) used proximity to and density of major roads from children’s homes.
