# Supplementary material for: Association between exposure to traffic-related air pollution and pediatric allergic diseases based on modeled air pollution concentrations and traffic measures in Seoul, Korea: a comparative analysis
Source: Environ Health. 2020 Jan 14;19:6. doi: 10.1186/s12940-020-0563-6 (PMC6961284; doi:10.1186/s12940-020-0563-6)
Supplement: Supplementary file 6 — Additional file 6: Figure S3. Scatter plots between predicted annual-average concentrations of NO2, PM10, or PM2.5 in 2010 and total length of major roads within 300 m buffers (Yi et al. 2017) at home addresses of 14,614 children in the Seoul Atopy Friendly School Project Survey in Seoul, Korea, for 2010 (red lines for non-linear relationships estimated by locally-weighted smoothing). [file 12940_2020_563_MOESM6_ESM.docx]

**
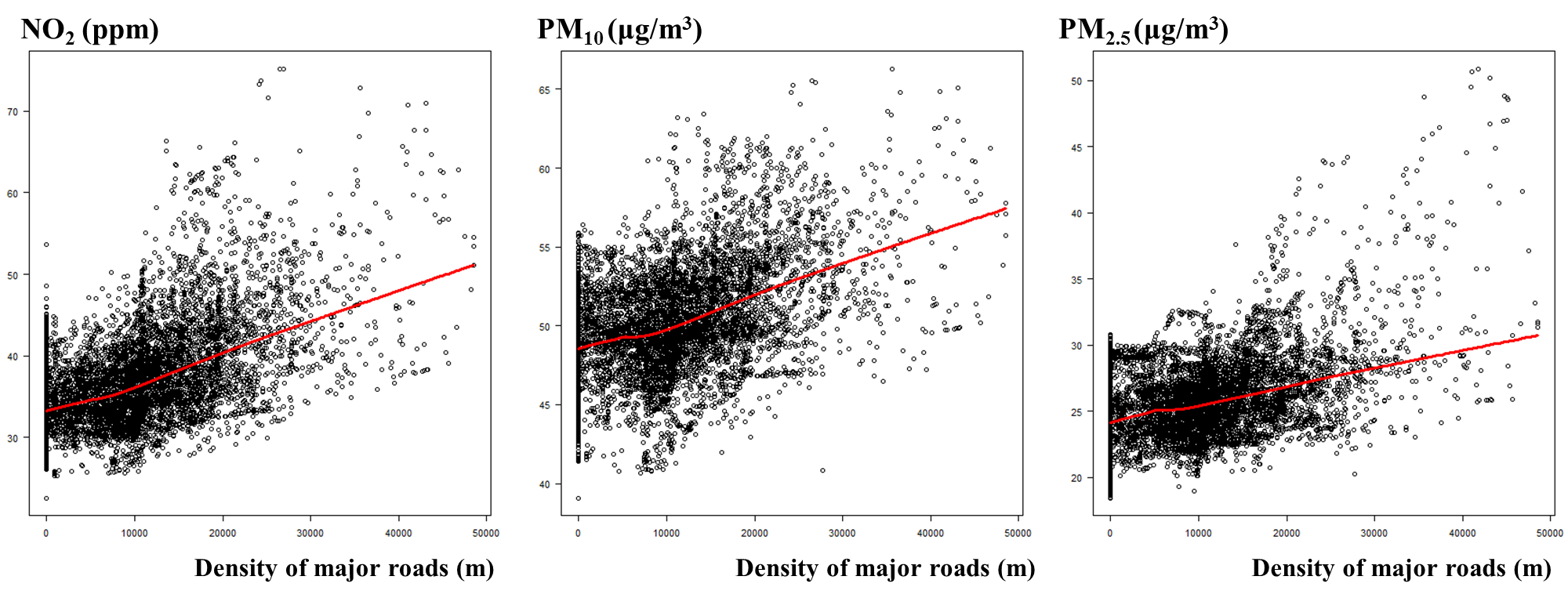
**

**Figure S3**. **Scatter plots between predicted annual-average concentrations of NO2, PM10, or PM2.5 in 2010 and total length of major roads within 300m buffers (Yi et al. 2017) at home addresses of 14,614 children in the Seoul Atopy Friendly School Project Survey in Seoul, Korea, for 2010 (red lines for non-linear relationships estimated by locally-weighted smoothing)**
