# Supplementary material for: Association between exposure to traffic-related air pollution and pediatric allergic diseases based on modeled air pollution concentrations and traffic measures in Seoul, Korea: a comparative analysis
Source: Environ Health. 2020 Jan 14;19:6. doi: 10.1186/s12940-020-0563-6 (PMC6961284; doi:10.1186/s12940-020-0563-6)
Supplement: Supplementary file 7 — Additional file 7: Figure S4. Odds ratios and 95% confidence intervals of symptoms and doctor-diagnoses of three allergic diseases for interquartile increases in individual-level annual average concentrations of NO2, PM10, and PM2.5 (4.7 ppm, 2.85 μg/m3, and 2.69 μg/m3, respectively) in 14,614 children at homes and schools from the Seoul Atopy Friendly School Project Survey in Seoul, Korea, for 2010. [file 12940_2020_563_MOESM7_ESM.docx]

**
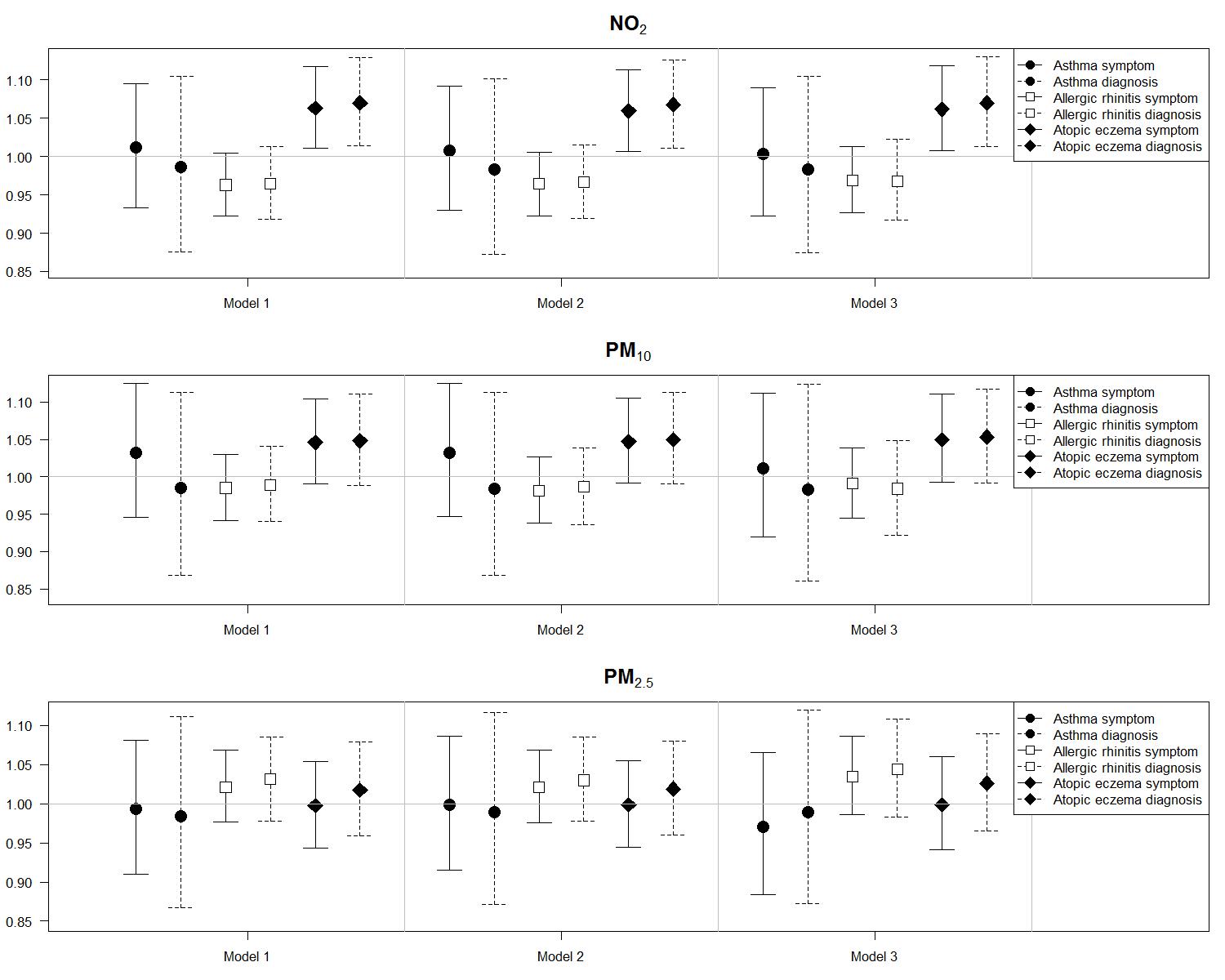
**

**Figure S4. Odds ratios and 95% confidence intervals of symptoms and doctor-diagnoses of three allergic diseases for interquartile increases in individual-level annual average concentrations of NO2, PM10, and PM2.5 (4.7 ppm, 2.85 ug/m3, and 2.69 ug/m3, respectively) in 14,614 children at homes and schools from the Seoul Atopy Friendly School Project Survey in Seoul, Korea, for 2010**

*Note*: We assessed individual exposures by using weighted averages with the ratio of 2 (home) to 1 (school)
