# Supplementary material for: Association between exposure to traffic-related air pollution and pediatric allergic diseases based on modeled air pollution concentrations and traffic measures in Seoul, Korea: a comparative analysis
Source: Environ Health. 2020 Jan 14;19:6. doi: 10.1186/s12940-020-0563-6 (PMC6961284; doi:10.1186/s12940-020-0563-6)
Supplement: Supplementary file 8 — Additional file 8: Table S4. Odds ratios and 95% confidence intervals of symptoms and doctor-diagnoses of three allergic diseases for interquartile increases in individual-level annual average concentrations of NO2, PM10, and PM2.5 (4.7 ppm, 2.85 μg/m3, and 2.69 μg/m3, respectively) in 14,614 children at homes and schools from the Seoul Atopy Friendly School Project Survey in Seoul, Korea, for 2010. [file 12940_2020_563_MOESM8_ESM.docx]

Table S4. Odds ratios and 95% confidence intervals of symptoms and doctor-diagnoses of three allergic diseases for interquartile increases in individual-level annual average concentrations of NO_2_, PM_10_, and PM_2.5_ (4.7 ppm, 2.85 ug/m^3^, and 2.69 ug/m^3^, respectively) in 14,614 children at homes and schools from the Seoul Atopy Friendly School Project Survey in Seoul, Korea, for 2010

| Response variable | Model | Explanatory variable | | |
| --- | --- | --- | --- | --- |
|  |  | NO_2_ | PM_10_ | PM_2.5_ |
| Asthma symptom  (N=12,518) | Model 1 | 1.01 (0.93 – 1.09) | 1.03 (0.95 – 1.12) | 0.99 (0.91 – 1.08) |
|  | Model 2 | 1.01 (0.93 – 1.09) | 1.03 (0.95 – 1.12) | 1.00 (0.92 – 1.09) |
|  | Model 3 | 1.00 (0.92 – 1.09) | 1.01 (0.92 – 1.11) | 0.97 (0.88 – 1.06) |
| Asthma  Diagnosis  (N=12,518) | Model 1 | 0.99 (0.88 – 1.11) | 0.99 (0.87 – 1.11) | 0.98 (0.87 – 1.11) |
|  | Model 2 | 0.98 (0.87 – 1.10) | 0.98 (0.87 – 1.11) | 0.99 (0.87 – 1.12) |
|  | Model 3 | 0.98 (0.87 – 1.11) | 0.98 (0.86 – 1.12) | 0.99 (0.87 – 1.12) |
| Rhinitis  Symptom  (N=12,518) | Model 1 | 0.96 (0.92 – 1.00) | 0.99 (0.94 – 1.03) | 1.02 (0.98 – 1.07) |
|  | Model 2 | 0.96 (0.92 – 1.01) | 0.98 (0.94 – 1.03) | 1.02 (0.98 – 1.07) |
|  | Model 3 | 0.97 (0.93 – 1.01) | 0.99 (0.94 – 1.04) | 1.03 (0.99 – 1.09) |
| Rhinitis  Diagnosis  (N=12,518) | Model 1 | 0.96 (0.92 – 1.01) | 0.99 (0.94 – 1.04) | 1.03 (0.98 – 1.09) |
|  | Model 2 | 0.97 (0.92 – 1.02) | 0.99 (0.94 – 1.04) | 1.03 (0.98 – 1.09) |
|  | Model 3 | 0.97 (0.92 – 1.02) | 0.98 (0.92 – 1.05) | 1.04 (0.98 – 1.11) |
| Eczema  Symptom  (N=14,614) | Model 1 | 1.06 (1.01 – 1.12) | 1.05 (0.99 – 1.10) | 1.00 (0.94 – 1.05) |
|  | Model 2 | 1.06 (1.01 – 1.11) | 1.05 (0.99 – 1.11) | 1.00 (0.94 – 1.05) |
|  | Model 3 | 1.06 (1.01 – 1.12) | 1.05 (0.99 – 1.11) | 1.00 (0.94 – 1.06) |
| Eczema  Diagnosis  (N=14,614) | Model 1 | 1.07 (1.01 – 1.13) | 1.05 (0.99 – 1.11) | 1.02 (0.96 – 1.08) |
|  | Model 2 | 1.07 (1.01 – 1.13) | 1.05 (0.99 – 1.11) | 1.02 (0.96 – 1.08) |
|  | Model 3 | 1.07 (1.01 – 1.13) | 1.05 (0.99 – 1.12) | 1.03 (0.97 – 1.09) |
