# Supplementary material for: Association between exposure to traffic-related air pollution and pediatric allergic diseases based on modeled air pollution concentrations and traffic measures in Seoul, Korea: a comparative analysis
Source: Environ Health. 2020 Jan 14;19:6. doi: 10.1186/s12940-020-0563-6 (PMC6961284; doi:10.1186/s12940-020-0563-6)
Supplement: Supplementary file 9 — Additional file 9: Table S5. Odds ratios (ORs) and 95% confidence intervals (95% CIs) of asthma symptoms and diagnoses for individual-level concentrations of NO2, PM10, PM2.5 by children with and without allergic rhinitis and atopic eczema diagnoses. [file 12940_2020_563_MOESM9_ESM.docx]

**Table S5. Odds ratios (ORs) and 95% confidence intervals (95% CIs) of asthma symptoms and diagnoses for individual-level concentrations of NO_2_, PM_10_, PM_2.5_ by children with and without allergic rhinitis and atopic eczema diagnoses**

| Asthma | Exposure | Presence of both allergic rhinitis and atopic eczema diagnosis | |
| --- | --- | --- | --- |
|  |  | Yes (N= 645) | No (N= 11,873)* |
| Symptom | NO_2_ | 1.02 (0.90 – 1.14) | 0.96 (0.84 – 1.09) |
|  | PM_10_ | 1.02 (0.89 – 1.17) | 1.01 (0.87 – 1.17) |
|  | PM_2.5_ | 0.99 (0.86 – 1.14) | 0.97 (0.84 – 1.12) |
| Diagnosis | NO_2_ | 0.99 (0.85 – 1.16) | 0.96 (0.79 – 1.17) |
|  | PM_10_ | 0.96 (0.81 – 1.15) | 1.03 (0.83 – 1.27) |
|  | PM_2.5_ | 0.99 (0.83 – 1.19) | 0.95 (0.77 – 1.17) |

* 11,873 participants with one or none of allergic disease diagnosis
