# Supplementary material for: Association between exposure to traffic-related air pollution and pediatric allergic diseases based on modeled air pollution concentrations and traffic measures in Seoul, Korea: a comparative analysis
Source: Environ Health. 2020 Jan 14;19:6. doi: 10.1186/s12940-020-0563-6 (PMC6961284; doi:10.1186/s12940-020-0563-6)
Supplement: Supplementary file 10 — Additional file 10: Table S6. Odds ratios (ORs) and 95% confidence intervals (95% CIs) of asthma symptoms and diagnoses for individual-level concentrations of NO2, PM10, PM2.5 by children with and without presence of at least one of allergic rhinitis and atopic eczema diagnosis. [file 12940_2020_563_MOESM10_ESM.docx]

**Table S6.** Odds ratios (ORs) and 95% confidence intervals (95% CIs) of asthma symptoms and diagnoses for individual-level concentrations of NO_2_, PM_10_, PM_2.5_ by children with and without presence of at least one of allergic rhinitis and atopic eczema diagnosis

| Asthma outcome | Exposure | Presence of at least one of allergic rhinitis and atopic eczema diagnosis | |
| --- | --- | --- | --- |
|  |  | Yes (N= 3,973) | No (N= 8,545)* |
| Asthma symptom | NO_2_ | 0.92 (0.74 – 1.15) | 0.99 (0.91 – 1.08) |
|  | PM_10_ | 0.86 (0.66 – 1.12) | 1.03 (0.93 – 1.14) |
|  | PM_2.5_ | 0.90 (0.70 – 1.15) | 0.99 (0.89 – 1.09) |
| Asthma diagnosis | NO_2_ | 1.03 (0.79 – 1.33) | 0.96 (0.84 – 1.09) |
|  | PM_10_ | 0.96 (0.72 – 1.27) | 0.99 (0.87 – 1.14) |
|  | PM_2.5_ | 1.09 (0.83 – 1.44) | 0.96 (0.83 – 1.10) |

*8,545 participants have none of allergic disease diagnosis
