# Supplementary material for: Association between exposure to traffic-related air pollution and pediatric allergic diseases based on modeled air pollution concentrations and traffic measures in Seoul, Korea: a comparative analysis
Source: Environ Health. 2020 Jan 14;19:6. doi: 10.1186/s12940-020-0563-6 (PMC6961284; doi:10.1186/s12940-020-0563-6)
Supplement: Supplementary file 11 — Additional file 11: Figure S5. Odds ratios and 95% confidence intervals of symptoms and doctor-diagnoses of three allergic diseases for interquartile increases in individual-level annual average concentrations of NO2 (6.46 ppm) in 14,614 children at homes stratified by regional and household socioeconomic status from the Seoul Atopy Friendly School Project Survey in Seoul, Korea, for 2010. [file 12940_2020_563_MOESM11_ESM.docx]

**
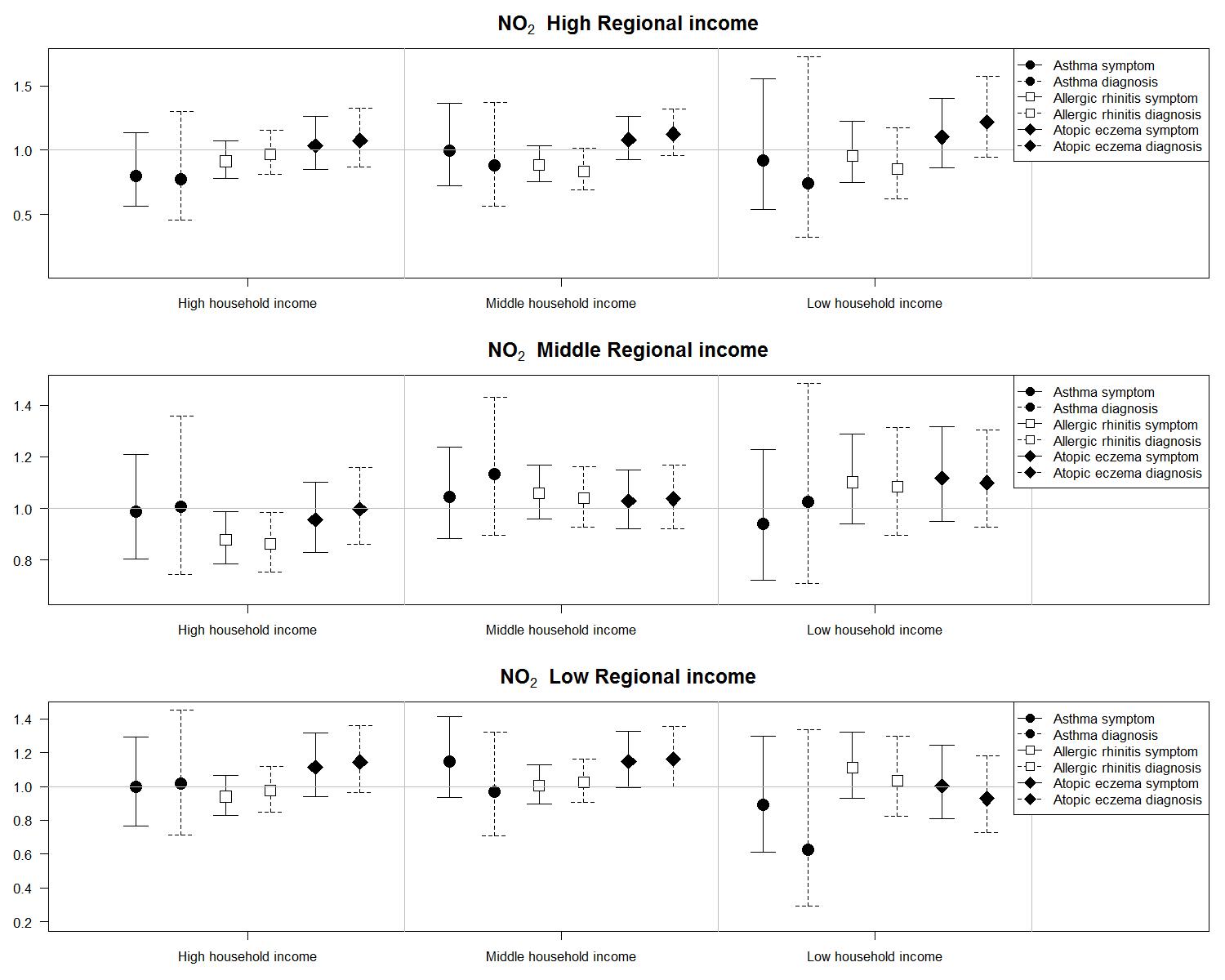
**

**Figure S5. Odds ratios and 95% confidence intervals of symptoms and doctor-diagnoses of three allergic diseases for interquartile increases in individual-level annual average concentrations of NO2 (6.46 ppm) in 14,614 children at homes stratified by regional and household socioeconomic status from the Seoul Atopy Friendly School Project Survey in Seoul, Korea, for 2010**
