# Supplementary material for: Association between exposure to traffic-related air pollution and pediatric allergic diseases based on modeled air pollution concentrations and traffic measures in Seoul, Korea: a comparative analysis
Source: Environ Health. 2020 Jan 14;19:6. doi: 10.1186/s12940-020-0563-6 (PMC6961284; doi:10.1186/s12940-020-0563-6)
Supplement: Supplementary file 13 — Additional file 13: Figure S7. Odds ratios and 95% confidence intervals of symptoms and doctor-diagnoses of three allergic diseases for interquartile increases in individual-level annual average concentrations of PM2.5 (3.63 μg/m3) in 14,614 children at homes stratified by regional and household socioeconomic status from the Seoul Atopy Friendly School Project Survey in Seoul, Korea, for 2010. [file 12940_2020_563_MOESM13_ESM.docx]

**
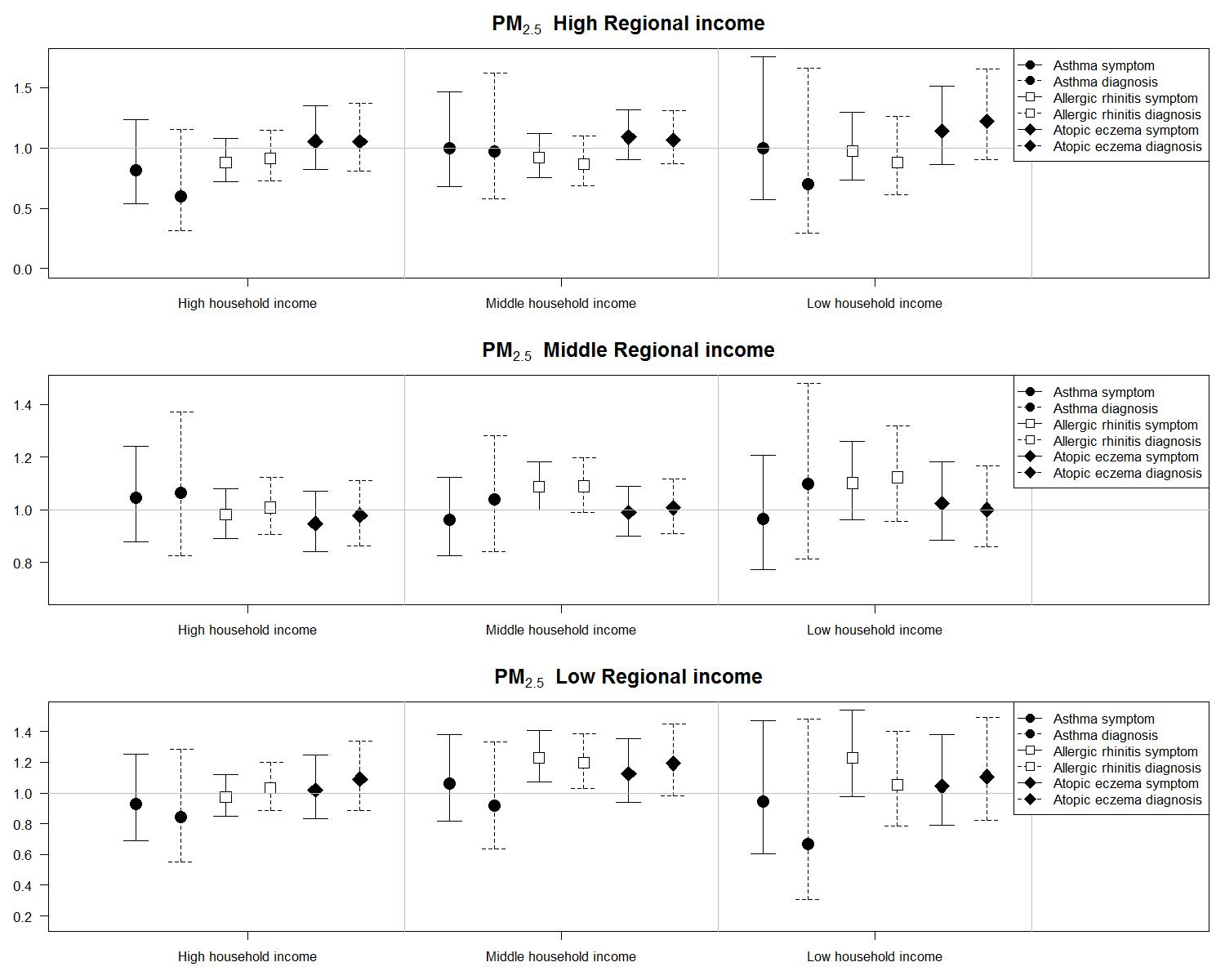
**

**Figure S7. Odds ratios and 95% confidence intervals of symptoms and doctor-diagnoses of three allergic diseases for interquartile increases in individual-level annual average concentrations of PM2.5 (3.63 ug/m3) in 14,614 children at homes stratified by regional and household socioeconomic status from the Seoul Atopy Friendly School Project Survey in Seoul, Korea, for 2010**
